# Discussion on the Pathology of Gas Gangrene

**Published:** 1919

**Authors:** 


**Discussion on the Pathology of Gas Gangrene.** By Dr. James McIntosh and Major J. W. McNee. *The Lancet,* December 14, 1918.

*Incidence and Bacteriology.* Gas gangrene appeared to have been almost unrecognized previous to the Crimean campaign. Its incidence among British troops, declining since the early months of this war, has lately been about 1 per cent.

The injured tissues seem to provide a suitable nidus for the growth of the anerobic bacteria present in all cultivated soils, and there is no doubt as to the cause of gas gangrene.

*Results of Investigations on Causation and Treatment.* This complication usually develops within the first 48 hours after injury, and its rapidity of spread is striking.

It was insisted by Wallace and Kenneth Taylor that gas gangrene was essentially a disease of muscle and never developed when muscle injury could be avoided. The spread of gas gangrene in a limb was found to be different whether or not there was an intact blood-supply. The essential factor in the spread was the passing up of a fluid substance in the sheaths of the muscle fibers, causing their death and their invasion by the anerobic bacilli. Recently experiments on rabbits have shown that the injection of organisms freshly isolated from a human case did not cause gas gangrene unless the muscle was first extensively injured.

In actual war wounds all the most favorable conditions for the development of gas gangrene were provided : the heavily manured soil, the short-range warfare of the trenches causing great laceration to muscle when a bone was struck, and the frequent infection of wounds by cloth, dirt, etc.

During 1916 and 1917 the dead and damaged muscle in which gas gangrene could develop was removed by operation as soon as possible. The majority of cases of gas gangrene occurred among men who had been brought to the casualty clearing stations over long distances, or who were too weak to endure operation. When gas gangrene is developing the blood pressure rises at first, and the medical officer may think that the man is recovering.

In the acute spreading gangrenes of limbs, B. Welchii was found by Bull to be the chief organism. An intense toxemia occurred only in the later stages just before death. Other forms of gas gangrene of intense toxemic type gave positive blood cultures of the Vibrion septique. Bull found an exotoxin produced by B. Welchii, and by injecting this obtained an antitoxic serum.

